# CT texture analysis of lung adenocarcinoma: can Radiomic features be surrogate biomarkers for EGFR mutation statuses

**DOI:** 10.1186/s40644-018-0184-2

**Published:** 2018-12-14

**Authors:** Dongdong Mei, Yan Luo, Yan Wang, Jingshan Gong

**Affiliations:** 1 Department of Radiology, Shenzhen People’s Hospital, the Second Clinical Medical College, Jinan University, Shenzhen, 518020 Guangdong China; 2 Department of Radiology and Biomedical Imaging, University of California San Francisco, 185 Berry St, Suite 350, San Francisco, CA 94107 USA

**Keywords:** Lung adenocarcinoma, Computed tomography, Radiomics, Epidermal growth factor receptor

## Abstract

**Objective:**

To investigate whether radiomic features can be surrogate biomarkers for epidermal growth factor receptor (EGFR) mutation statuses.

**Materials and methods:**

Two hundred ninety six consecutive patients, who underwent CT examinations before operation within 3 months and had EGFR mutations tested, were enrolled in this retrospective study. CT texture features were extracted using an open-source software with whole volume segmentation. The association between CT texture features and EGFR mutation statuses were analyzed.

**Results:**

In the 296 patients, there were 151 patients with EGFR mutations (51%). Logistic analysis identified that lower age (Odds Ratio[OR]: 0.968,95% confidence interval [CI]:0.946~0.990, *p* = 0.005) and a radiomic feature named GreyLevelNonuniformityNormalized (OR: 0.012, 95% CI:0.000~0.352, *p* = 0.01) were predictors for exon 19 mutation; higher age (OR: 1.027, 95%CI:1.003~1.052,*p* = 0.025), female sex (OR: 2.189, 95%CI:1.264~3.791, *p* = 0.005) and a radiomic feature named Maximum2DDiameterColumn (OR: 0.968, 95%CI:0.946~0.990], p = 0.005) for exon 21 mutation; and female sex (OR: 1.883,95%CI:1.064~3.329, *p* = 0.030), non-smoking status (OR: 2.070, 95%CI:1.090~3.929, *p* = 0.026) and a radiomic feature termed SizeZone NonUniformityNormalized (OR: 0.010, 95% CI:0.0001~0.852, *p* = 0.042) for EGFR mutations. Areas under the curve (AUCs) of combination with clinical and radiomic features to predict exon 19 mutation, exon 21 mutation and EGFR mutations were 0.655, 0.675 and 0.664, respectively.

**Conclusion:**

Several radiomic features are associated with EGFR mutation statuses of lung adenocarcinoma. Combination with clinical files, moderate diagnostic performance can be obtained to predict EGFR mutation status of lung adenocarcinoma. Radiomic features might harbor potential surrogate biomarkers for identification of EGRF mutation statuses.

## Introduction

Lung cancer is the leading cause of cancer-related death for both men and women [1]. Non–small cell lung cancer (NSCLC) accounts for 85–90% of lung cancers, while lung adenocarcinoma is the most diagnosed histological subtype of NSCLC [2]. In the past decade treatment for NSCLC has evolved from the use of cytotoxic chemotherapy to personalized treatment based on molecular alterations, especially in the treatment of patients with epidermal growth factor receptor (EGFR) mutations [3]. Small molecule tyrosine kinase inhibitors had demonstrated a higher response rate to patients with EGFR mutation (60–80%) than those with EGFR wild type or unknown mutation status (10–20%) [4]. Comparing with EGFR wild type, longer progression-free survival (PFS) and higher objective radiographic response rates were also observed in patients with mutated EGFR lung cancer [5]. Therefore, acknowledge of EGFR mutation status of lung adenocarcinomas is essential for personalized therapy. As most solid tumors, lung adenocarcinomas also show widespread molecular heterogeneity. Regional heterogeneity in the distribution of mutations of lung adenocarcinoma genomes is also observed [3]. The heterogeneity is a critical barrier to the development of precision medicine approaches because the standard approach to tumor sampling, often invasive needle biopsy, is unable to fully capture the spatial state of the tumor [6]. On the other hand, molecular analysis is often expensive and is based on adequate tumor cells. At some times, rebiopsy is needed.

CT is the most common modality, which is used by clinicians to diagnosis, assessment of stage and treatment response of lung cancers. It can be accessible commonly and repeat at low price and little invasion. Several CT features had been revealed to be associated with EGFR mutation status [7]. Conventional analysis of medical imaging relies on visual assessment of radiologists. As the medical imaging is regarded as pictures, abundant data features beyond the naked eye ability may be abandoned. Radiomics, which uses computers to extract a large number of quantitative features from image data to explore biologic properties of lesions, and subsequently to analyze these features for decision making, has emerged as a promising technique to identify gene phenotype in several kinds of tumors. The latter is termed as radiogenomic. Using radiogenomic, Liu et al. disclosed that mutant EGFR status could be predicted by a set of 5 radiomic features [8]. As the lung cancer is of high mutation burden, EGFR mutation can occur from exon 18 to exon 21. The sensitivity of EGFR mutant tumors to molecule tyrosine kinase inhibitors is also different among the genotype of EGFR [9].

Exon 19 mutations are associated with a higher response rate and longer survival after treatment with tyrosine kinase inhibitors than exon 21 mutation [10, 11]. Exon 19 and 21 mutations consist of 90% of EGFR mutation in lung adenocarcinomas, identifying these two kinds of mutations is essential for personalized treatment [12]. We hypothesize that the differences between EGFR mutations may result into microstructure alternations. The aim of this study is to assess whether radiomic features using CT texture analysis can identify lung adenocarcinomas with EGFR exon 19 mutation and 21 mutation.

## Materials and methods

### Patients

This retrospective study was approved by the Medical Ethics Committee of Shenzhen People’s Hospital and informed consent was waived. From January 2011 to October 2017, 306 consecutive patients with pathologic proved lung adenocarcinoma were chosen from the database of our hospital. The inclusion criteria were: (1) with thoracic CT before operation within 3 months; (2) with results of EGFR mutation status; (3)with available clinical data.

### Ct

CT scans were performed by 16-slice (Brilliance 16, Philips health system, Cleveland, USA) and 128-slice (Brilliance iCT, Philips health system, Cleveland, USA) CT scanner, without iv contrast medium administration. Collimation of 16 × 1.5 mm was used for the 16-slice scanner, while collimation of 128 × 0.625 mm was used for the 128-slice scanner, both with automatic adjustment of tube current. Images were reconstructed with slice thickness of 2 mm and 1 mm increment for 16-slice scanner, while for 128-slice scanner with slice thickness of 1.5 mm and 0.75 mm increment, both with Y-sharp filter. All the image data were read from the picture archive and communication system (PACS)..

### Tumor segmentation and Radiomic features extraction

**Fig. 1:**
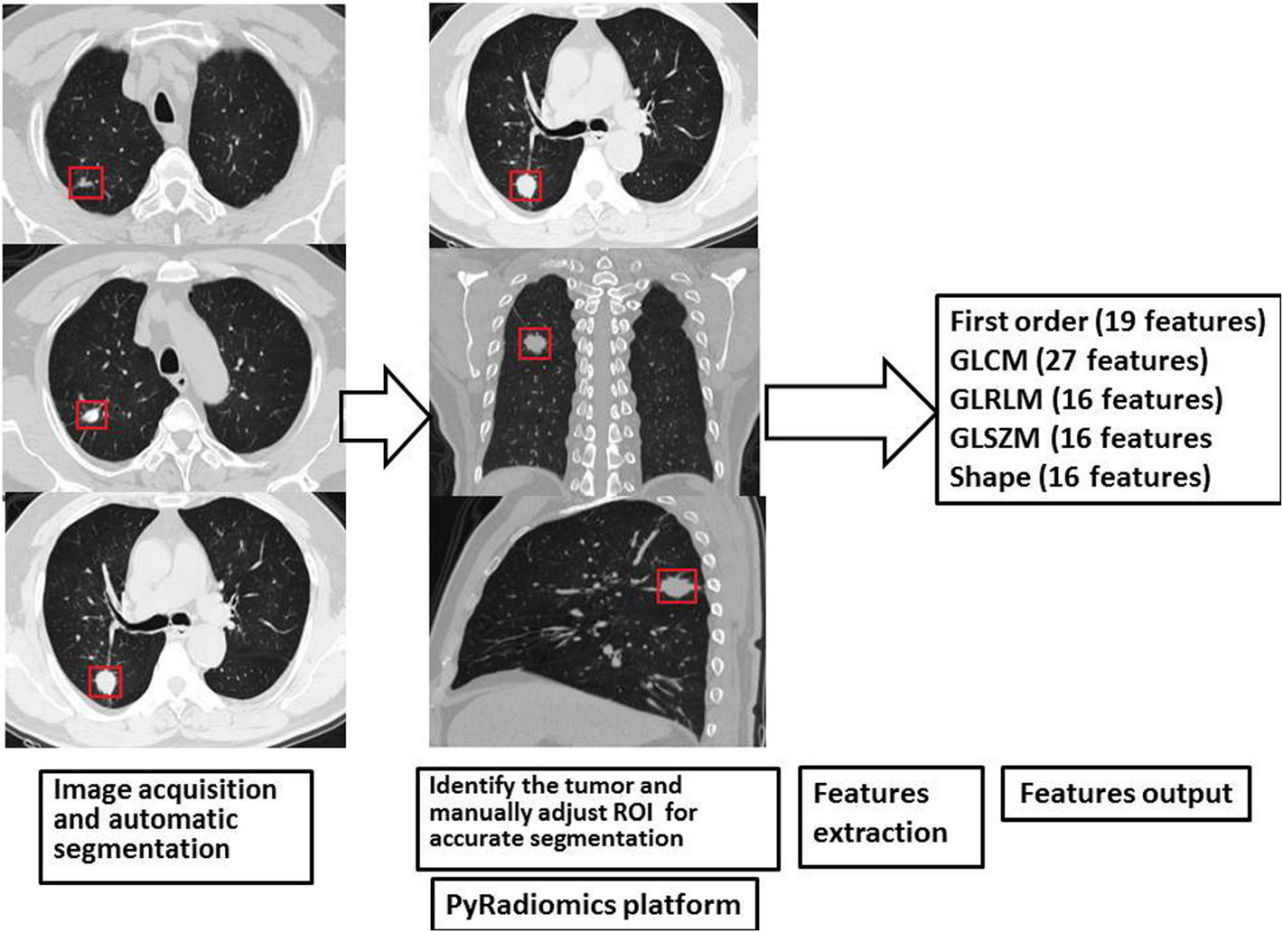
The process of PyRadiomics.The process includes the software automatically segments three lesions in the right lung at first, a radiologist identifies the cancer according to surgery record and makes some manual adjustments for accurate segmentation, then radiomic features are extracted and outputted for analysis

The three-dimensional volume CT image data were transferred into a computer and the radiomic features were extracted using an open-source software called PyRadiomics, which are available at http://www.radiomics.io/pyradiomics.html. The operation processing can be found in the reference [13]. The software can identify and segment pulmonary lesions automatically (Fig. 1). Manual adjustment of region of interest (ROI) was made for accurate segmentation by a radiologist with 3-year-experience in thoracic radiology. If there were multiple pulmonary lesions, the radiologist identified the tumor according to pathological recorder and surgical markers. Ninety-four texture features, including first order features (19 features), gray-level-co-occurrence matrix (GLCM) features (27 features), gray-level-run-length matrix (GLRLM) features (16 features), gray-level size zone matrix (GLSZM) features (16 features) and shape features (16 features), are extracted from the marketed lesions. The definition of these radiomic features are available at http://pyradiomics.readthedocs.io/en/latest/features.html. First order features describe the distribution of voxel intensities within the ROI using common and basic metrics. GLCM features describe the second-order joint probability function of an image region constrained by a mask. GLRLM features quantify gray level runs, which are defined as the length in number of pixels, of consecutive pixels that have the same gray level value. GLSZM features quantify gray level zones in an image. A gray level zone is defined as a the number of connected voxels that share the same gray level intensity. Shape features include descriptors of the three-dimensional size and shape of the ROI.

### Statistical analysis

The statistical analysis was performed with IBM SPSS statistics version 24. A 2-sided *P* value of < .05 was regarded as statistically significant. Univariate analysis was performed first. Fisher exact test and the Kruskal-Wallis test were used for categorical and continuous variables, respectively. Then, all the clinical characteristics and radiomics features with statistical significance were entered logistic regression analysis. Receiver operating characteristic (ROC) curves were constructed and the Area under the curve (AUC) was calculated for predicting EGFR mutation status with radiomics features and the combination of radiomics features and clinical presentations, respectively.

## Results

### Patients’ clinical features and EGFR mutations

**Fig. 2:**
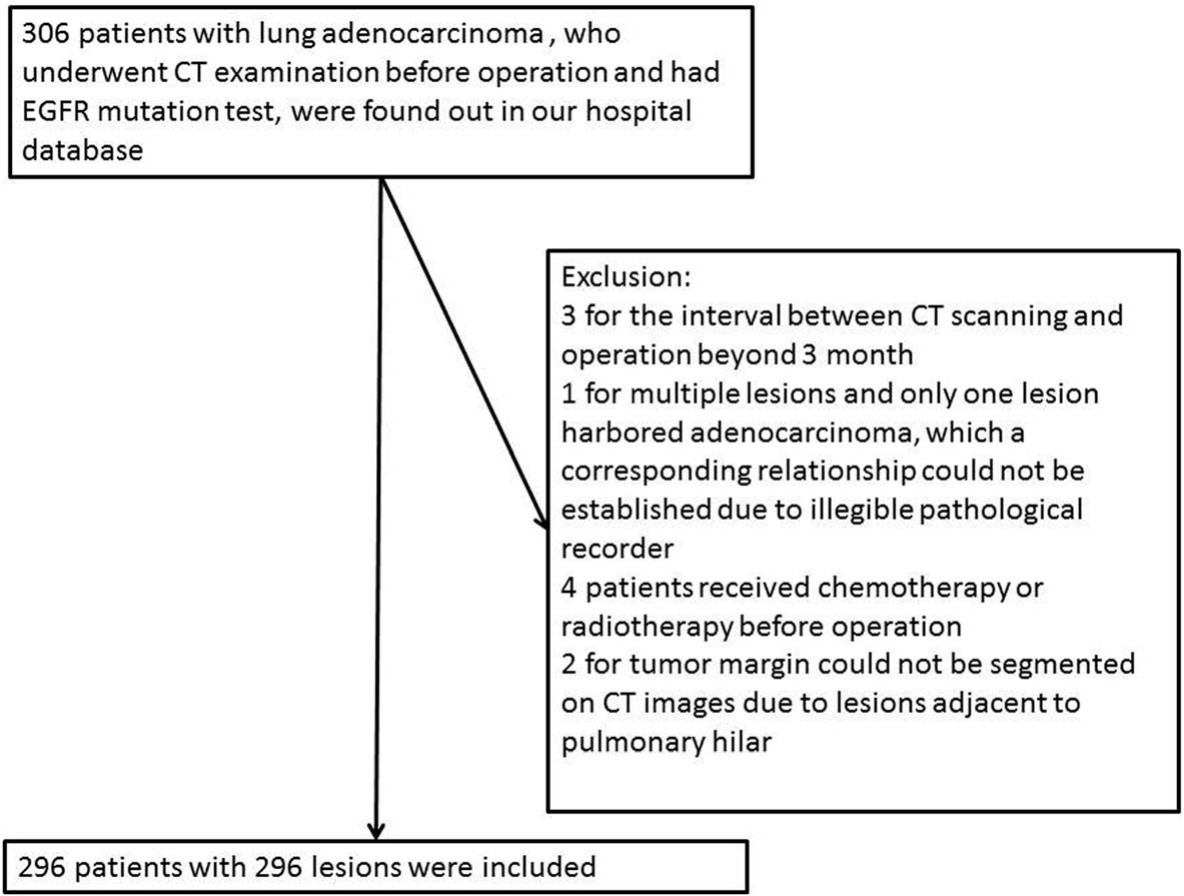
The workflow diagram shows selection of study population and exclusion criteria

The final study population included 296 patients who fulfilled the inclusion criteria, ten patients were excluded (3 for the interval between CT scanning and operation beyond 3 month; 1 for multiple lesions and only one lesion harbored adenocarcinoma, which a corresponding relationship could not be established due to illegible pathological recorder; 4 patients received chemotherapy or radiotherapy before operation; 2 for tumor margin could not be segmented on CT images due to lesions adjacent to pulmonary hilar). A flow chart of the study population is presented in Fig. 2. In the 296 patients, there were 5 tumors with EGFR exon 18 mutation, 66 with exon 19 mutation, 7 with exon 20 mutation and 78 with exon 21 mutation. 5 tumors harbored 2 exon mutations. No patients had more than 2 exon mutations. Therefore, there were 151 patients with EGFR mutations with a prevalence of 51%. The patients’ clinical features were presented in Table 1. EGFR mutations were more common in females and nonsmoker with statistical significance.

**Table 1:** Clinical features of patients with lung adenocarcinoma

	EGRF mutation (*n* = 151)	EGFR wild type (*n* = 145)	*P* value
Age	56.69 ± 12.30	60.43 ± 12.23	0.604
gender			0.000
male	59	93	
female	92	52	
Smoking status			0.000
smoker	27	59	
Non-smoker	124	86	

### Texture analysis and diagnostic performance

Due to only 5 and 7 tumors with exon 18 and 20 exon mutations, the present study focused on exon 19 and 21 mutations, respectively. Then all the exon mutations were taken into account and the patients were dichotomized into with and without EGFR mutation. At univariate analysis, 21 radiomic features and age were significantly associated with exon 19 mutation; 21 radiomic features, smoking status and gender with exon 21 mutation; 2 radiomic features, smoking status and gender with EGFR mutations. The variables with statistical significance at univariate analysis and Odds Ratio at logistic analysis were presented in Table 2.

**Table 2:** Variables with statistical significance at univariate analysis and logistic regression

	variable	p value	logistic regression	
			Odds Ratio (95% confidence interval)	*p* value
Exon 19 mutation	Age	0.004	0.968(0.946~0.990)	0.005
	Entropy	0.016		
	InterquartileRange	0.004		
	Kurtosis	0.011		
	MeanAbsoluteDeviation	0.015		
	RobustMeanAbsoluteDeviation	0.005		
	StandardDeviation	0.021		
	Uniformity	0.023		
	Variance	0.021		
	ClusterTendency	0.017		
	Correlation	0.014		
	DifferenceEntropy	0.047		
	Entropy	0.025		
	Imc1	0.03		
	Imc2	0.006		
	SumEntropy	0.014		
	SumSquares	0.018		
	SumVariance	0.017		
	GrayLevelNonUniformityNormalized	0.008	0.012(0.000~0.352)	0.01
	GrayLevelVariance	0.031		
	RunEntropy	0.026		
	ShortRunEmphasis	0.06		
Exon 21 mutation	age	0.04	1.027(1.003~1.052)	0.025
	Smoking status	0.005		
	Gender	0.004	2.189(1.264~3.791)	0.005
	Maximum	0.015		
	Range	0.02		
	Autocorrelation	0.048		
	ClusterProminence	0.06		
	HighGrayLevelRunEmphasis	0.04		
	ShortRunHighGrayLevelEmphasis	0.036		
	GrayLevelNonUniformityNormalized	0.034		
	GrayLevelVariance	0.039		
	SizeZoneNonUniformity	0.013		
	SizeZoneNonUniformityNormalized	0.012		
	SmallAreaEmphasis	0.014		
	LeastAxis	0.009		
	MajorAxis	0.043		
	Maximum2DDiameterColumn	0.012	0.968(0.946~0.990)	0.005
	Maximum2DDiameterRow	0.066		
	Maximum2DDiameterSlice	0.019		
	Maximum3DDiameter	0.025		
	MinorAxis	0.021		
	SurfaceArea	0.02		
	SurfaceVolumeRatio	0.011		
	Volume	0.017		
EGFR mutation	Gender	0.00016	1.883(1.064~3.329)	0.030
	Smoking status	0.00015	2.070(1.090~3.929)	0.026
	SizeZoneNonUniformityNormalized	0.026	0.010(0.0001~0.852)	0.042
	SmallAreaEmphasis	0.037		

**Fig. 3:**
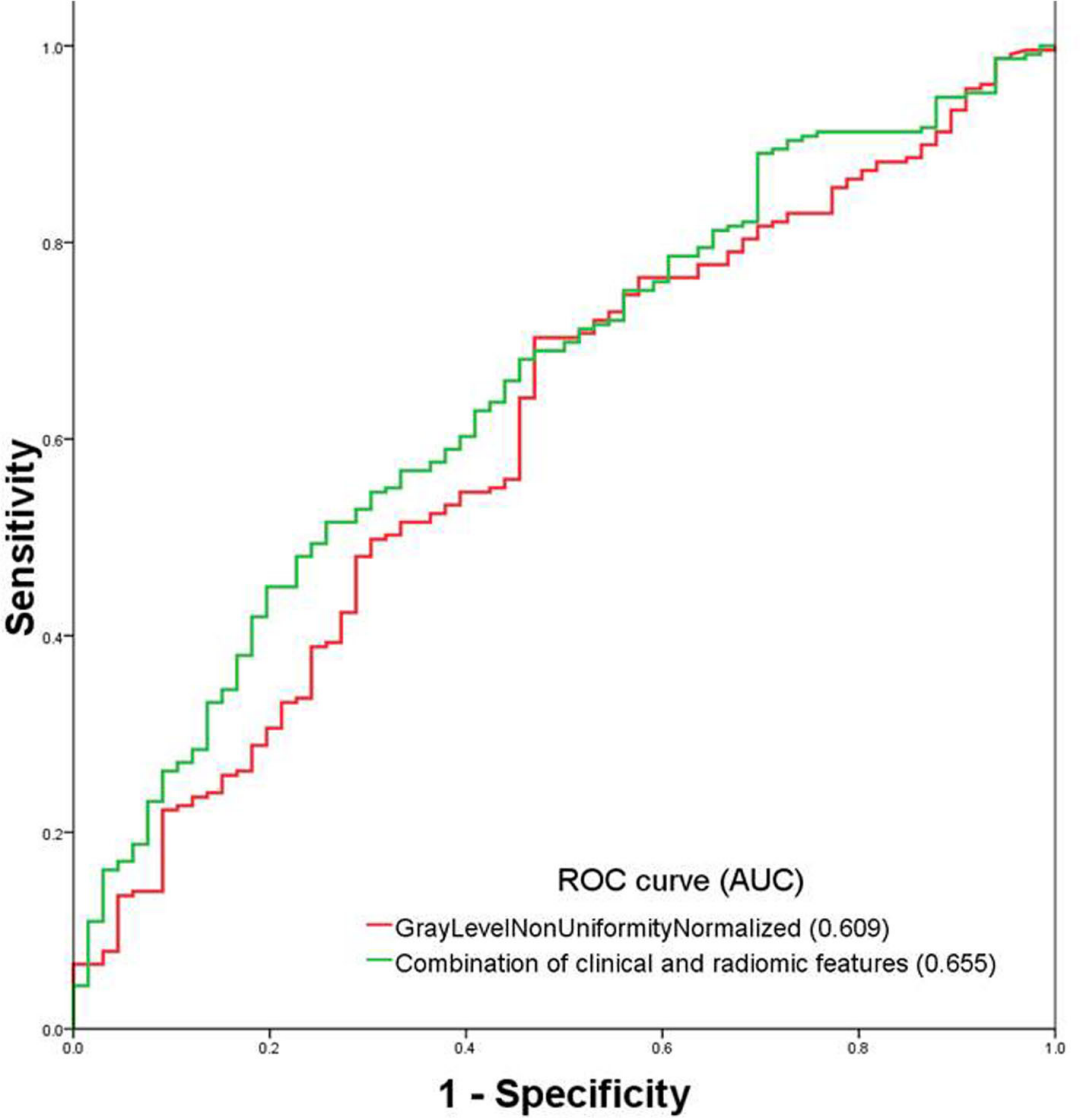
ROC curve of the radiomic GLCM feature named GreyLevelNonuniformityNormalized and combination of radiomic feature and clinical feature to predict exon 19 mutation

Regarding to exon 19 mutation, logistic analysis showed that lower age (Odds Ratio[OR]: 0.968, 95% confidence interval [CI]:0.946~0.990, *p* = 0.005) and a radiomic GLCM feature named GreyLevelNonuniformityNormalized (OR: 0.012, 95%CI:0.000~0.352, *p* = 0.01) were the risk factors. The Area under the curve (AUC) of the radiomic feature to predict exon 19 mutation was 0.609. After combining age, AUC reached 0.655 (Fig. 3).

**Fig. 4:**
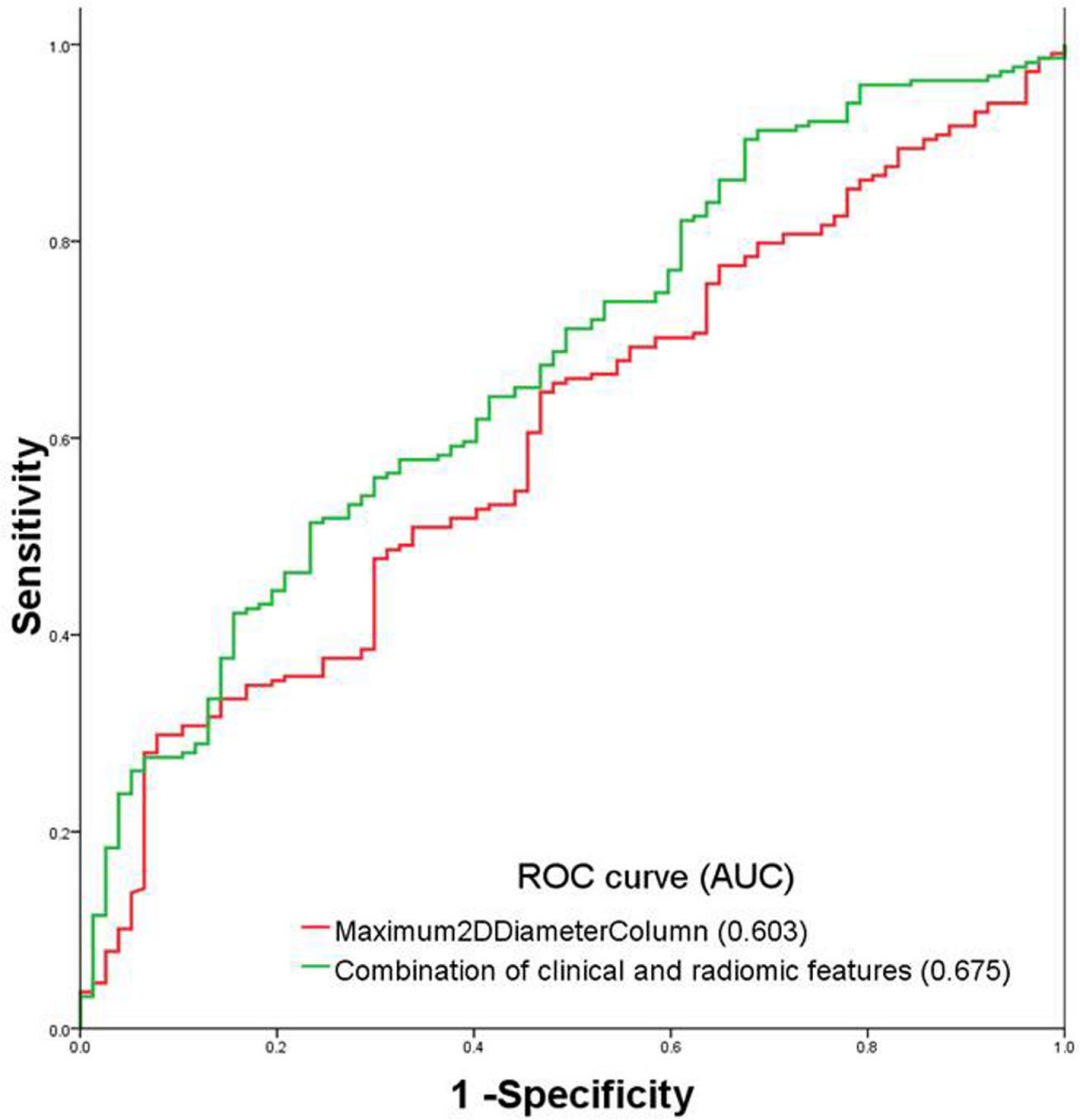
ROC curve of the radiomic shape feature named Maximum2DDiameterColumn and combination of radiomic feature and clinical feature to predict exon 21 mutation

Higher age (OR: 1.027, 95%CI:1.003~1.052, *p* = 0.025), female sex (OR: 2.189 95%CI:1.264~3.791, *p* = 0.005) and a radiomic shape feature named Maximum2DDiameterColumn (OR: 0.968,95%CI:0.946~0.990, p = 0.005) were found to be associated with exon 21 mutation at logistic analysis. The AUC of the radiomic feature only and combination of clinical presentations to predict exon 21 mutation was 0.603 and 0.675, respectively (Fig. 4).

**Fig. 5:**
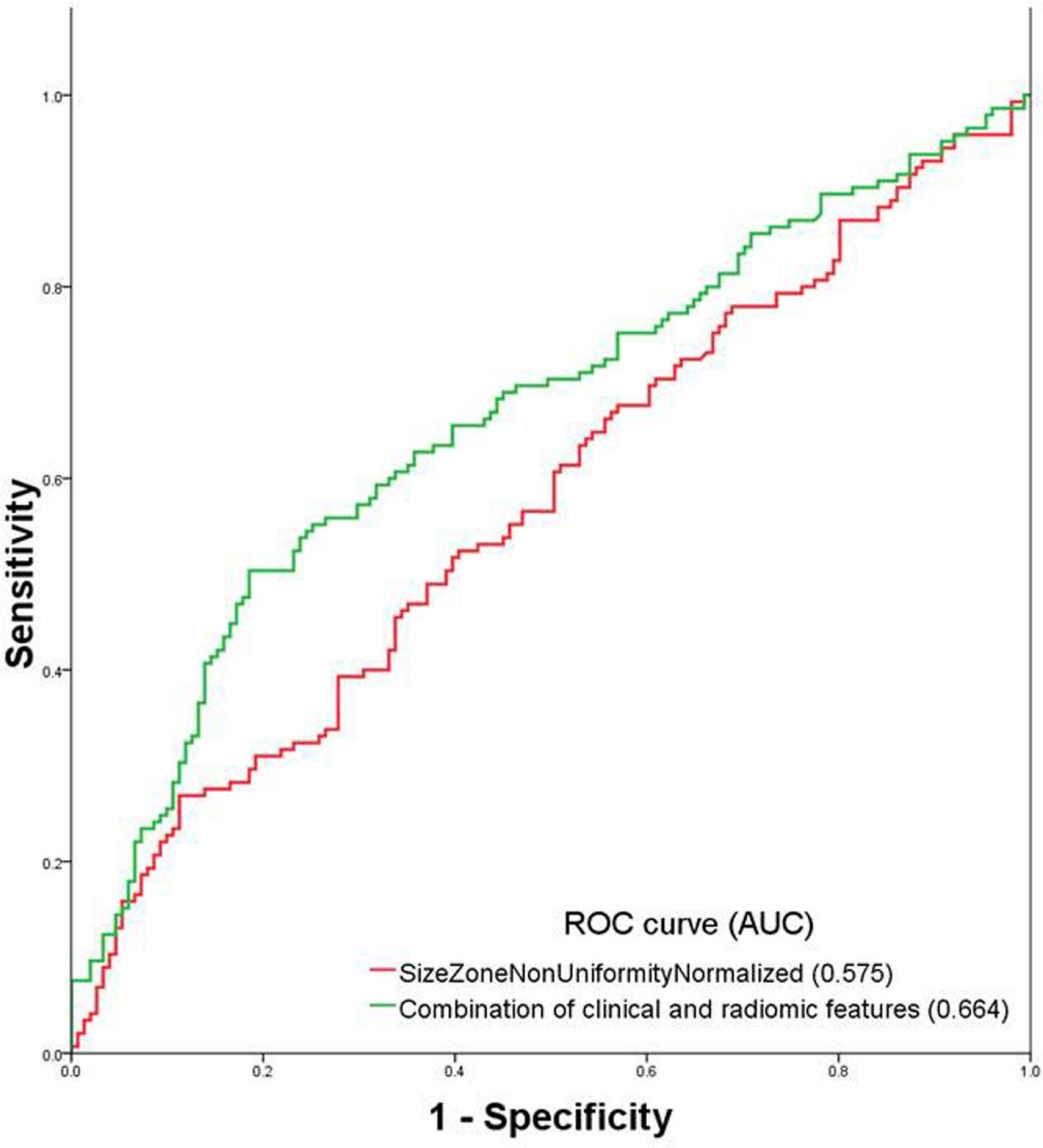
ROC curve of the radiomic GLSZM feature termed SizeZoneNonUniformityNormalized and combination of radiomic feature and clinical feature to predict EGFR mutation

When the patients were dichotomized into with and without EGFR mutation, female sex (OR: 1.883, 95%CI:1.064~3.329, *p* = 0.030), non-smoking status (OR: 2.070, 95%CI:1.090~3.929, *p* = 0.026) and a radiomic GLSZM feature termed SizeZoneNonUniformityNormalized (OR: 0.010, 95% CI:0.0001~0.852, *p* = 0.042) were found to be risk factors for EGFR mutations. The AUC of the radiomic feature only and combination of clinical presentations to predict EGFR mutation was 0.575 and 0.664, respectively (Fig. 5).

## Discussion

In this study, we attempt to assess the association of radiomics features with EGFR exon 19 and 21 mutations of lung adenocarcinomas, respectively. A separate analysis of EGFR exon 19 and 21 mutations may facilitate personalized treatment of lung adenocarcinomas. Moderate diagnostic performance was obtained from the combination of radiomic features and clinical presentations to predict EGFR exon 19 and 21 mutations of lung adenocarcinomas.

Recently development of personalized treatment to lung carcinoma has attracted more attention to EGFR mutations. Patients with EGFR mutations, especially with exon 19 mutation, showed better prognosis for tyrosine kinase inhibitors treatment. Therefore, acknowledge of EGFR mutation status is essential for personalized treatment. Epidemiology studies have shown that several clinical characteristics, such as female, nonsmoker and East Asian origination, were associated with EGFR mutation [14, 15]. At present study, female and nonsmoker were also found to be associated with EGFR mutation, we also identified that age was associated with different exon mutations. Patients with exon 19 mutation tended to be younger (OR = 0.968), while patients with exon 21 mutation tended to be older (OR = 1.027). This phenomenon has not been reported before. One explanation might be racial characteristics. Another explanation might be selection bias introduced by the retrospective study nature. No clinical features can be used to predict EGFR mutation precisely. Histopathological samples are most common used for probing EGFR mutation status. Because lung cancers are very heterogeneous, histopathological analysis may introduce sampling bias. Especially in some patients, only biopsy samples can be obtained, which may contain a few tumor cells. Therefore, an ease accessible modality which can evaluate the whole tumor at once examination is needed.

CT, as the most common modality for lung cancer, can obtain abundant structure information of the whole tumor at one scanning. In a study, Rizzo and colleagues investigated the association between conventional CT features and EGFR, ALK, KRAS mutations in non-small cell lung cancer [7]. They found that some traditional CT features, including air bronchogram, pleural retraction, small lesion size and absence of fibrosis, were associated with EGFR mutations in non-small cell lung cancer. Even though in the study, a AUC of 0.8235 was obtained after combining CT features and clinical characteristics to predict EGFR mutations, exon mutations of EGFR were not analyzed separately and abundant digital information of CT images beyond perceivable by the radiologists’ naked eyes were not included. Radiomics, termed as high throughput extraction of medical imaging characteristics from digital imaging data, has emerged recently and shows its promising ability for an improved decision support [16–19]. Radiomics also shows the ability to serve as a bridge between medical imaging and precise medicine [20]. In lung cancer, several studies had demonstrated the possibility to use radiomic features as biomarkers for patients’ outcome or genetic characteristics. To the best of knowledge, the relationship between radiomic features and EGFR, especially regarding exon mutations separately, had not well established. In a study, Ozkan [21] and colleagues explored the association between CT gray-level texture features and EGFR mutation status in a small patient group of 25 patients with EGFR mutation and 20 patients with EGFR wild type. Although they identified several CT gray-level texture features were associated with EGFR mutations, the small sample size might introduce selection bias. In another study, Liu et al. included 298 patients and obtained an AUC of 0.709 for predicting EGFR mutation when radiomic features were combined with clinicopathological characteristics. In the study, exon mutations were not treated separately. In this study, a similar sample size as Liu et al. was explored and exon 19 and exon 21 mutations were analyzed respectively. For clinical characteristics, we found that female and nonsmoker status were associated with high prevalence of EGFR mutations and exon 21 mutation, while younger patients were like to have exon 19 mutation. Different radiomic features were associated with EGFR mutation status. Using logistic regression, we identified several clinical characteristics and radiomic features were risk factors for EGFR mutation status. Moderate diagnostic performance was obtained after combination of clinical and radiomic risk factors (AUC of 0.655, 0.675 and 0.664 for exon 19 mutations, exon 21 mutations and the whole exon mutations, respectively). Due to lacking standard, different studies use different methods for texture analysis and many software are developed in-house. Therefore, the reproduction and comparison are difficult, even impossible. In this study, we use an open-source software called PyRadiomic, which is developed from Python and the all the source code and documents can be accessible at http://www.radiomics.io/pyradiomics.html. We wish our data can be comparable with other studies or future studies using the same software.

Our preliminary study showed that radiomic features extracted from CT images might be promising biomarkers to predict EGFR mutations of lung cancer in vivo. Because lung cancers are high heterogeneous, gene-expression profiling based on tissue specimens may have sampling errors, especially for biopsy specimens. Radiomic features can overcome the limitation to capture intratumoral heterogeneity in a non-invasion three-dimension manner. Harnessing these radiomic features can aid decision making in clinical practice, such as guiding biopsy and treatment selection. Therefore, radiomics signatures from CT images might be a powerful tool for precision diagnosis and treatment of lung cancer.

Several limitations presented at this study. At first, it was retrospective study and there was patient selection bias. Second, the tumor identification was determined by one radiologist who known the operation recorder when there were more than one pulmonary lesion. Therefore, the operator-dependence might be introduced. In addition, traditional radiological findings were not included in present study. Rizzo and colleagues reported that some traditional radiological findings were associated with genotype of lung cancers [7]. Therefore, combination of the traditional radiological findings and radiomic features might improve the diagnosis performance to predict EGFR mutation statuses. Another limitation was that age was found to be associated with different exon mutations at present study, but the underlying reason failed to be identified.

## Conclusion

In summary, this study showed that several radiomic features were associated with EGFR mutation statuses in lung adenocarcinomas. Even though after combination of clinical characteristics and radiomic features, only moderate diagnostic performance was obtained, radiomic features might harbor potential surrogate biomarkers for identification of EGFR mutations. Further radiogenomic studies with large sample size are needed to nail down those features, which can predict the EGFR mutation in lung adenocarcinomas prospectively.

## Abbreviations

AUC: Area under the curve
CI: Confidence interval
EGFR: Epidermal growth factor receptor
GLCM: Gray-level-co-occurrence matrix
GLRLM: Gray-level-run-length matrix
GLSZM: Gray-level size zone matrix
NSCLC: Non–small cell lung cancer
OR: Odds Ratio
PACS: Picture archive and communication system
PFS: Progression-free survival
ROC: Receiver operating characteristic.
ROI: Region of interest.
